# Global prevalence of *Cryptosporidium* spp. in pigs: a systematic review and meta-analysis

**DOI:** 10.1017/S0031182023000276

**Published:** 2023-05

**Authors:** Yuancai Chen, Huikai Qin, Yayun Wu, Huiyan Xu, Jianying Huang, Junqiang Li, Longxian Zhang

**Affiliations:** College of Veterinary Medicine, Henan Agricultural University, Zhengzhou 450002, P. R. China

**Keywords:** *Cryptosporidium*, meta-analysis, pig, prevalence

## Abstract

*Cryptosporidium* spp. are significant opportunistic pathogens causing diarrhoea in humans and animals. Pigs are one of the most important potential hosts for *Cryptosporidium*. We evaluated the prevalence of *Cryptosporidium* in pigs globally using published information and a random-effects model. In total, 131 datasets from 36 countries were included in the final quantitative analysis. The global prevalence of *Cryptosporidium* in pigs was 16.3% (8560/64 809; 95% confidence interval [CI] 15.0–17.6%). The highest prevalence of *Cryptosporidium* in pigs was 40.8% (478/1271) in Africa. Post-weaned pigs had a significantly higher prevalence (25.8%; 2739/11 824) than pre-weaned, fattening and adult pigs. The prevalence of *Cryptosporidium* was higher in pigs with no diarrhoea (12.2%; 371/3501) than in pigs that had diarrhoea (8.0%; 348/4874). Seven *Cryptosporidium* species (*Cryptosporidium scrofarum*, *Cryptosporidium suis*, *Cryptosporidium parvum*, *Cryptosporidium muris*, *Cryptosporidium tyzzeri*, *Cryptosporidium andersoni* and *Cryptosporidium struthioni*) were detected in pigs globally. The proportion of *C. scrofarum* was 34.3% (1491/4351); the proportion of *C. suis* was 31.8% (1385/4351) and the proportion of *C. parvum* was 2.3% (98/4351). The influence of different geographic factors (latitude, longitude, mean yearly temperature, mean yearly relative humidity and mean yearly precipitation) on the infection rate of *Cryptosporidium* in pigs was also analysed. The results indicate that *C. suis* is the dominant species in pre-weaned pigs, while *C. scrofarum* is the dominant species in fattening and adult pigs. The findings highlight the role of pigs as possible potential hosts of zoonotic cryptosporidiosis and the need for additional studies on the prevalence, transmission and control of *Cryptosporidium* in pigs.

## Graphical abstract

**Figure:**
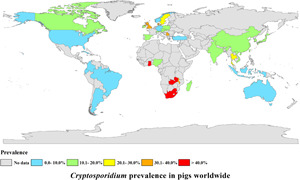

## Introduction

*Cryptosporidium* is an opportunistic zoonotic parasite found worldwide that infects many vertebrate hosts and typically causes self-limiting diarrhoea in humans and livestock (Kotloff, 2017; Hatam-Nahavandi *et al*., 2019). *Cryptosporidium* is commonly found in the intestines of humans and animals and is transmitted by the fecal–oral route (Bouzid *et al*., 2013). Children, immunodeficient individuals and newborn animals are among the groups that are susceptible to *Cryptosporidium* infection (Checkley *et al*., 2015). Among animals susceptible to *Cryptosporidium*, pigs are considered as one of the main reservoir hosts (Qi *et al*., 2020). There are no effective vaccines that can prevent cryptosporidiosis in humans or livestock (Dumaine *et al*., 2020).

Globally, the first report of 3 pig cases of cryptosporidiosis was in 1977 (Kennedy *et al*., 1977). Pigs with cryptosporidiosis are characterized by diarrhoea, vomiting, dehydration, reduced daily gain and a lower feed conversion rate (Vítovec and Koudela, 1992; Quílez *et al*., 1996; Enemark *et al*., 2003), and the parasites mainly live in the intestinal tract and gallbladder (Fleta *et al*., 1995). There is considerable genetic variation in the genus *Cryptosporidium*; there are 44 known species, and more than 120 genotypes of *Cryptosporidium* have been identified (Ryan *et al*., 2021). Thirteen different *Cryptosporidium* species/genotypes have been isolated in pigs, namely *Cryptosporidium scrofarum* (previously *Cryptosporidium* pig genotype II), *Cryptosporidium suis* (previously *Cryptosporidium* pig genotype I), *Cryptosporidium muris*, *Cryptosporidium parvum*, *Cryptosporidium tyzzeri* (previously *Cryptosporidium* mouse genotype I), *Cryptosporidium hominis*, *Cryptosporidium meleagridis*, *Cryptosporidium felis*, *Cryptosporidium andersoni*, *Cryptosporidium struthioni*, *Cryptosporidium* rat genotype, *Cryptosporidium* sp. Eire w65.5 and unknown *Cryptosporidium* genotype from pig slurry (Němejc *et al*., 2013*b*; Wang *et al*., 2021, 2022). *Cryptosporidium scrofarum* and *C. suis* infections account for more than 90% of cryptosporidiosis in pigs (Feng *et al*., 2018). Cryptosporidiosis in pigs does not always cause clinical symptoms, and cases of human infection with *C. scrofarum* and *C. suis* suggest that these 2 *Cryptosporidium* species may be zoonotic (Kvác *et al*., 2009*c*; Moore *et al*., 2016; Sannella *et al*., 2019). However, their pathogenicity and infectivity to humans are not well understood; so, they remain a potential threat to human health.

The global pig population was estimated at 952.6 million in 2020 (https://www.fao.org/). In animal husbandry, cryptosporidiosis causes huge economic losses due to weight loss in young animals, stunted growth and reduced production in adult animals (Pumipuntu and Piratae, 2018). Pigs are also animals that humans often contact directly or indirectly. Therefore, we performed a systematic review and meta-analysis to assess the global prevalence of *Cryptosporidium* in pigs. The potential risk factors including region, age and geographical and climatic factors were also analysed. The results describe the distribution characteristics of *Cryptosporidium* species in different age groups of pigs, and provide a basis for the prevention and control of *Cryptosporidium* infections.

## Materials and methods

### Search strategy and selection criteria

We used 5 literature databases (PubMed, Web of Science, the China National Knowledge Infrastructure, VIP Chinese Journals Database and Wanfang Data) to search for studies on the global prevalence of *Cryptosporidium* in pigs. All published studies on *Cryptosporidium* in pigs from 31 September 2022 onwards were included. We searched the 2 English databases with the term ‘*Cryptosporidium*’, ‘Cryptosporidiosis’ cross-referenced with ‘pig’, ‘swine’, ‘hog’, ‘wart’, ‘warthog’, ‘Phacochoerus’, ‘Suidae’, ‘boar’ or ‘piglet’. In the 3 Chinese databases, ‘*Cryptosporidium*’ (Chinese) and ‘pig’ (Chinese) were used as keywords. We conducted analyses in accordance with the preferred reporting items for systematic reviews and meta-analyses (PRISMA) statement and the PRISMA 2009 checklist (Table S1). The articles for which full text was not available, the first author was not contacted for more research information and/or statistics.

The following clauses were used as the criteria for article exclusion:

1. the purpose of the study was not the prevalence of *Cryptosporidium* in pigs;
2. the total number of pigs tested and the number of pigs that tested positive were not provided;
3. the testing method was not clearly described;
4. the sample was a mixture of specimens from multiple pig feces;
5. the study sample size was less than 20;
6. the study was a review or a case report.

### Quality assessment

We used established methods to evaluate the quality of the studies (Guyatt *et al*., 2008). Studies with scores of 0 or 1 point were classified as low quality, studies with scores of 2 or 3 points were classified as medium quality, and studies with scores of 4 or 5 points were classified as high quality. A study scored 1 point if it included one of the following items:

1. a clear research goal;
2. a clearly defined research period;
3. a sample size of greater than 200;
4. a clear detection method;
5. analysis involving 3 or more influencing factors.

### Data extraction

Two authors (Y. C. and H. Q.) separately screened all titles, abstracts and full texts and independently extracted the data. Disagreements were resolved by discussion with Y. W. Y. C. and H. Q. extracted information, including the first author, publication date, country, sampling time, detection method, total samples, positive samples, prevalence, study quality and *Cryptosporidium* species (Table S2).

### Statistical analysis

All data were analysed using Stata version 14.0 (https://www.stata.com). Due to high heterogeneity (*I*^2^ > 50%, *P* < 0.1) of the data, the random-effects model was used for the meta-analysis. To investigate the potential sources of heterogeneity, sensitivity analysis, subgroup analysis and meta-regression analysis were performed on the extracted data. If a study involved multiple detection methods for *Cryptosporidium*, the molecular results in the analysis were the first choice. We used sensitivity analysis to test the stability of the data, and the overall study was evaluated using forest plots. We evaluated the effect of selected studies on the pooled prevalence by excluding single studies sequentially (Wang *et al*., 2018*b*). Publication bias of the study was evaluated using a funnel plot and Egger's tests (Egger *et al*., 1997). The following potential sources of heterogeneity were examined: region (Asia compared to other regions), age (post-weaned compared to the other age groups), presence or absence of diarrhoea (diarrhoea compared to non-diarrhoea) and *Cryptosporidium* species (*C. scrofarum* compared to the other species).

The global longitude and latitude span was large, and there were significant geographical differences. The data related to geographic factors were obtained from the National Oceanic and Atmospheric Administration (NOAA, https://gis.ncdc.noaa.gov/maps/ncei/cdo/monthly). We also used subgroup analysis and meta-regression analysis to evaluate the impact of geographical risk factors, including latitude (30°–60° *vs* others), longitude (<−60° *vs* others), mean yearly temperature (5–10 °C *vs* others), mean yearly relative humidity (<60% *vs* others), mean yearly precipitation (0–400 mm *vs* others).

## Results

### Characteristics of studies

**Fig. 1.**
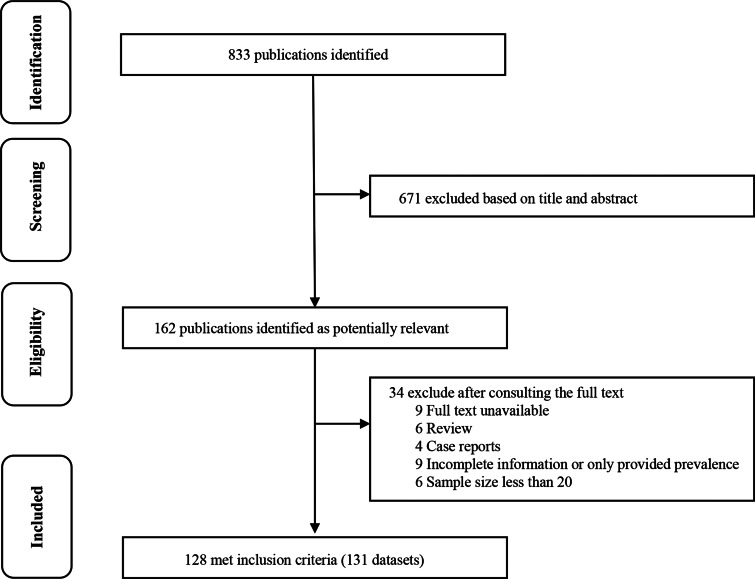
Flow diagram of the selection of eligible studies.

A total of 833 publications were initially identified. After screening of the title and abstract, 162 potentially relevant articles were selected for full text search. Of these, 6 were review studies, 9 had incomplete information or only provided prevalence, 6 had sample sizes less than 20, 4 were case reports and 9 lacked full text. In total, 128 publications (including 131 datasets) were of sufficient quality and were considered suitable for meta-analysis (Fig. 1).

**Fig. 2.**
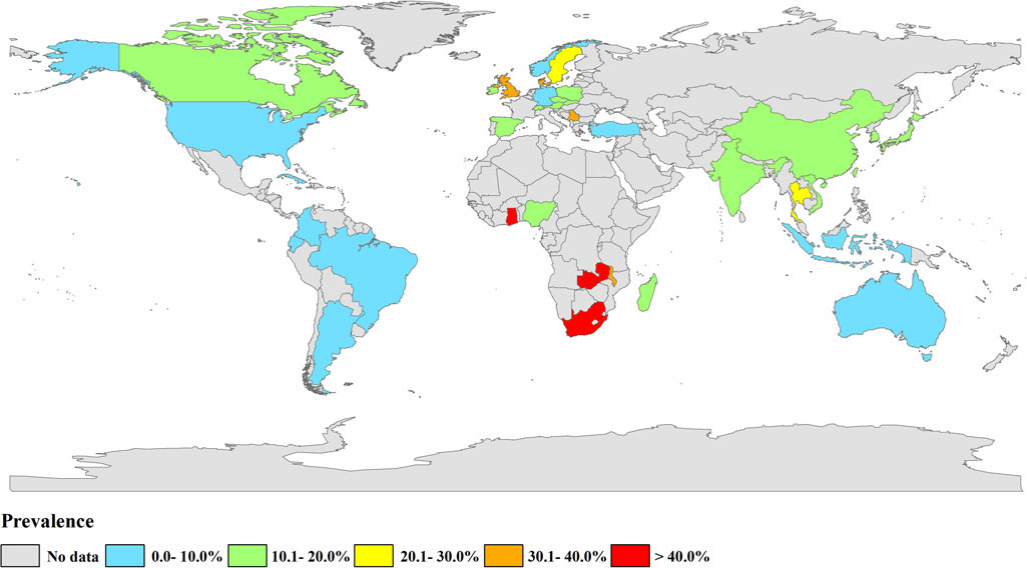
Map of *Cryptosporidium* infection in pigs across the world. Prevalence ranges are shown in different colours. [The figure was designed using Arcgis 10.2, and the original vector diagram imported in Arcgis was adapted from Natural Earth (http://www.naturalearthdata.com).]

The selected studies came from 36 countries (Fig. 2, Table 1). A total of 71 datasets originated from Asia [China (*n* = 54), India (*n* = 2), Indonesia (*n* = 1), Japan (*n* = 6), Korea (*n* = 3), Thailand (*n* = 1), Turkey (*n* = 1), Vietnam (*n* = 3)]. A total of 30 datasets were from countries in Europe [Austria (*n* = 1), Czech Republic (*n* = 6), Denmark (*n* = 2), Germany (*n* = 2), Ireland (*n* = 1), Norway (*n* = 1), Poland (*n* = 2), Serbia (*n* = 1), Slovak Republic (*n* = 2), Spain (*n* = 8), Sweden (*n* = 1), Switzerland (*n* = 1) and the UK (*n* = 1)]. Eight datasets were from countries in Africa [Ghana (*n* = 1), Madagascar (*n* = 1), Malawi (*n* = 1), Nigeria (*n* = 3), South Africa (*n* = 1), Zambia (*n* = 1)]. A total of 10 datasets were from countries in North America [Canada (*n* = 4), Trinidad (*n* = 1), the USA (*n* = 4), Cuba (*n* = 1)]. Eight datasets were from South America [Argentina (*n* = 1), Brazil (*n* = 4), Colombia (*n* = 2), Ecuador (*n* = 1)]. Four datasets were from countries in Oceania [Australia (*n* = 4)] (Tables 1 and 2). Pre-weaned pigs were described in 48 datasets, post-weaned pigs were described in 63 datasets, fattening pigs were described in 48 datasets and adult pigs were described in 53 datasets. Most datasets lacked information on pig health status. Diarrhoea in pigs was reported in 14 datasets, and no diarrhoea in pigs was reported in 10 datasets (Table 2).

**Table 1.** Estimated pooled prevalence of *Cryptosporidium* infection by country/region

Country/regions	No. of studies	Region	No. tested	No. positive	% Prevalence	% (95% CI)
China	54	Asia	34650	4066	11.7	11.4–12.1
India	2	Asia	1195	131	11.0	9.2–12.7
Indonesia	1	Asia	205	13	6.3	3.0–9.7
Japan	6	Asia	2039	283	13.9	12.4–15.4
Korea	3	Asia	1582	212	13.4	11.7–15.1
Thailand	1	Asia	245	51	20.8	15.7–25.9
Turkey	1	Asia	238	21	8.8	5.2–12.5
Vietnam	3	Asia	961	164	17.1	14.7–19.4
Austria	1	Europe	44	8	18.2	6.3–30.0
Czech Republic	6	Europe	6939	943	13.6	12.8–14.4
Denmark	2	Europe	2093	745	35.6	33.5–37.6
Germany	2	Europe	1714	6	0.4	0.1–0.6
Ireland	1	Europe	342	39	11.4	8.0–14.8
Norway	1	Europe	684	57	8.3	6.3–10.4
Poland	2	Europe	295	57	19.3	14.8–23.9
Serbia	1	Europe	260	89	34.2	28.4–40.0
Slovak Republic	2	Europe	156	19	12.2	7.0–17.4
Spain	8	Europe	2378	325	13.7	12.3–15.0
Sweden	1	Europe	222	56	25.2	19.5–31.0
Switzerland	1	Europe	125	18	14.4	8.2–20.6
UK	1	Europe	308	119	38.6	33.2–44.1
UK/Ireland	1	Europe	56	25	44.6	31.2–58.1
Ghana	1	Africa	200	154	77.0	71.1–82.9
Madagascar	1	Africa	40	8	20.0	7.0–33.0
Malawi	1	Africa	92	30	32.6	22.8–42.4
Nigeria	3	Africa	632	118	18.7	15.6–21.7
South Africa	1	Africa	90	72	80.0	71.6–88.4
Zambia	1	Africa	217	96	44.2	37.6–50.9
Canada	4	North America	2593	302	11.6	10.4–12.9
Trinidad	1	North America	275	54	19.6	14.9–24.4
USA	4	North America	922	42	4.6	3.2–5.9
Cuba	1	North America	90	9	10.0	3.7–16.3
Argentina	1	South America	520	47	9.0	6.6–11.5
Brazil	4	South America	499	15	3.0	1.5–4.5
Colombia	2	South America	628	57	9.1	6.8–11.3
Ecuador	1	South America	26	2	7.7	0.0–18.7
Australia	4	Oceania	1254	107	8.5	7.0–10.1

**Table 2.** Pooled prevalence of *Cryptosporidium* infection in pigs across the world

	Number of datasets	Total samples	Positive samples	Prevalence % (95% CI)	Heterogeneity			Univariate meta-regression		Correlation analysis
					*χ*^2^	*P* value	*I*^2^	*P* value	Coefficient (95% CI)	Adj *R*^2^
Region								0.381	−0.183 (−0.593 to 0.228)	−0.18%
Asia	71	41 115	4941	14.8 (13.0–16.5)	4585.09	<0.001	98.5%			
Europe	30	15 616	2506	18.3 (14.4–22.2)	3167.63	<0.001	99.1%			
Africa	8	1271	478	40.8 (20.6–61.0)	528.56	<0.001	98.7%			
North America	10	3880	407	13.6 (8.6–18.7)	373.54	<0.001	97.6%			
South America	8	1673	121	7.1 (3.6–10.5)	47.05	<0.001	87.2%			
Oceania	4	1254	107	9.3 (2.2–16.4)	110.28	<0.001	97.3%			
Age								<0.001	0.606 (0.270–0.942)	5.77%
Pre-weaned	48	11 370	1061	12.0 (9.9–14.0)	964.35	<0.001	95.6%			
Post-weaned	63	11 824	2739	25.8 (21.8–29.8)	3198.35	<0.001	98.1%			
Fattening	48	8815	1186	17.4 (14.8–20.0)	1189.03	<0.001	96.3%			
Adult	53	9658	980	12.7 (10.4–15.1)	1224.19	<0.001	96.7%			
Diarrhoea								0.367	−0.323 (−1.051 to 0.405)	−0.66%
Yes	14	4874	348	8.0 (5.6–10.3)	113.84	<0.001	88.6%			
No	10	3501	371	12.2 (8.4–15.9)	94.20	<0.001	90.4%			
Species								0.002	0.775 (0.302–1.248)	7.91%
*C. scrofarum*	50	23 168	1491	7.9 (6.9–8.8)	1505.18	<0.001	96.7%			
*C. suis*	43	25 036	1385	4.7 (3.8–5.6)	1060.81	<0.001	96.0%			
Other^a^	19	6701	155	1.8 (1.2–2.5)	147.42	<0.001	87.8%			
**Total**	131	64 809	8560	16.3 (15.0–17.6)	10 445.02	<0.001	98.8%			

a Including *C. parvum*, *C. muris*, *C. tyzzeri*, *C. andersoni*, *C. struthioni*, *Cryptosporidium* spp.

**Table 3.** Extracted data from included studies for molecular methods of *Cryptosporidium* species

Country/region	Author (year of publication)	No. of positive samples	Species (no.)
Argentina	De Felice *et al*. (2020)	47	*C. scrofarum* (12), *C. suis* (7)
Australia	Johnson *et al*. (2008)	64	*C. scrofarum* (32), *C. suis* (13)
Australia	Ng *et al*. (2011)	3	*C. scrofarum* (3)
Australia	Ryan *et al*. (2003)	39	*C. scrofarum* (14), *C. suis* (14)
Austria	Němejc *et al*. (2013*b*)	8	*C. scrofarum* (6), *C. suis* (5)^a^
Brazil	Fiuza *et al*. (2011)	2	*C. scrofarum* (2)
Canada	Budu-Amoako *et al*. (2012)	163	*C. scrofarum* (69), *C. suis* (42), *C. tyzzeri* (1), *C. parvum* (2)
Canada	Farzan *et al*. (2011)	68	*C. scrofarum* (21), *C. suis* (1), *C. muris* (3), *C. parvum* (31)
China	Chen *et al*. (2011)	800	*C. scrofarum* (12), *C. suis* (63)^a^
China	Feng *et al*. (2020)	15	*C. scrofarum* (15)
China	Han *et al*. (2018)	17	*C. scrofarum* (16), *C. suis* (1)
China	Lam *et al*. (2022)	23	*C. scrofarum* (22), *C. suis* (1)
China	Li *et al*. (2016)	6	*C. scrofarum* (6)
China	Li *et al*. (2017)	3	*C. scrofarum* (3)
China	Li *et al*. (2018*a*)	24	*C. scrofarum* (24)
China	Li *et al*. (2022)	8	*C. scrofarum* (6), *C. suis* (2)
China	Lin *et al*. (2015)	44	*C. scrofarum* (2), *C. suis* (42)
China	Liu *et al*. (2021)	2	*C. parvum* (2)
China	Qi *et al*. (2020)	143	*C. scrofarum* (51), *C. suis* (90), *C. parvum* (2)
China	Wang *et al*. (2010)	111	*C. scrofarum* (14), *C. suis* (94)
China	Wang *et al*. (2018*a*)	28	*C. scrofarum* (10), *C. suis* (18)
China	Wang *et al*. (2019)	41	*C. struthioni* (41)
China	Wang *et al*. (2022)	57	*C. scrofarum* (46), *C. suis* (11)
China	Yang *et al*. (2020)	64	*C. scrofarum* (64)
China	Yao *et al*. (2020)	101	*C. scrofarum* (90), *C. suis* (4), *C. parvum* (7)
China	Yin *et al*. (2011)	16	*C. scrofarum* (16)
China	Yin *et al*. (2013)	79	*C. scrofarum* (65), *C. suis* (14)
China	Zhang *et al*. (2013)	63	*C. scrofarum* (41), *C. suis* (40)^a^
China	Zhang *et al*. (2020)	9	*C. scrofarum* (7), *C. suis* (2)
China	Zheng *et al*. (2019)	23	*C. scrofarum* (11), *C. suis* (12)
China	Zou *et al*. (2017)	70	*C. scrofarum* (70)
Czech Republic	Kvác *et al*. (2009*a*)	38	*C. scrofarum* (36), *C. suis* (15)^a^, *C. parvum* (2)
Czech Republic	Kvác *et al*. (2009*b*)	87	*C. scrofarum* (23), *C. suis* (44), *C. muris* (2)
Czech Republic	Němejc *et al*. (2012)	32	*C. scrofarum* (19), *C. suis* (25)^a^
Czech Republic	Němejc *et al*. (2013*a*)	353	*C. scrofarum* (208), *C. suis* (224)^a^, *C. parvum* (1), *C. muris* (3)
Czech Republic	Němejc *et al*. (2013*b*)	39	*C. scrofarum* (26), *C. suis* (25)^a^
Czech Republic	Vítovec *et al*. (2006)	394	*C. suis* (394)
Denmark	Langkjaer *et al*. (2007)	395	*C. scrofarum* (133), *C. suis* (50)
Denmark	Petersen *et al*. (2015)	350	*C. scrofarum* (38), *C. suis* (18)
Germany	Wieler *et al*. (2001)	4	*C. parvum* (4)
Indonesia	Resnhaleksmana *et al*. (2021)	13	*C. parvum* (13)
Ireland	Zintl *et al*. (2007)	39	*C. scrofarum* (11), *C. suis* (14), *C. parvum* (2), *C. muris* (1)
Japan	Katsuda *et al*. (2006)	14	C. parvum (14)
Japan	Yui *et al*. (2014*b*)	112	*C. scrofarum* (24), *C. suis* (21)^a^
Poland	Němejc *et al*. (2013*b*)	11	*C. scrofarum* (10), *C. suis* (3)^a^
Poland	Rzeżutka *et al*. (2014)	46	*C. scrofarum* (40), *C. suis* (7), *C. parvum* (1), *Cryptosporidium* spp. (1)
Slovak Republic	Danišová *et al*. (2016)	16	*C. scrofarum* (11), *C. suis* (2), *C. muris* (3)^a^, *C. andersoni* (1)
Slovak Republic	Němejc *et al*. (2013*b*)	3	*C. scrofarum* (1), *C. suis* (2)
Spain	García-Presedo *et al*. (2013)	35	*C. scrofarum* (19), *C. suis* (5), *C. parvum* (3)
Spain	Rivero-Juarez *et al*. (2020)	27	*C. scrofarum* (26), *C. suis* (1)
Spain	Suárez-Luengas *et al*. (2007)	32	*C. scrofarum* (16), *C. suis* (10)
Sweden	Pettersson *et al*. (2020)	56	*C. scrofarum* (36), *C. suis* (17), *C. parvum* (2)
Switzerland	Schubnell *et al*. (2016)	18	*C. scrofarum* (8), *C. suis* (4)
Thailand	Thathaisong *et al*. (2020)	51	*C. scrofarum* (42), *C. suis* (9)
UK\Ireland	Xiao *et al*. (2006)	25	*C. scrofarum* (11), *C. suis* (16), *C. muris* (1)
USA	Atwill *et al*. (1997)	12	*C. parvum* (12)
USA	Rodriguez-Rivera *et al*. (2016)	6	*C. scrofarum* (3), *C. suis* (1)
Vietnam	Iwashita *et al*. (2021)	2	*C. suis* (2)
**Total**		**4351**	***C. scrofarum*** (**1491), *C. suis* (1385), *C. parvum* (98), *C. struthioni* (41), *C. muris* (13), *C. tyzzeri* (1), *C. andersoni* (1), *Cryptosporidium* spp. (1)**

a Mixed infection

### *Cryptosporidium* infection in pigs by region

**Fig. 3.**
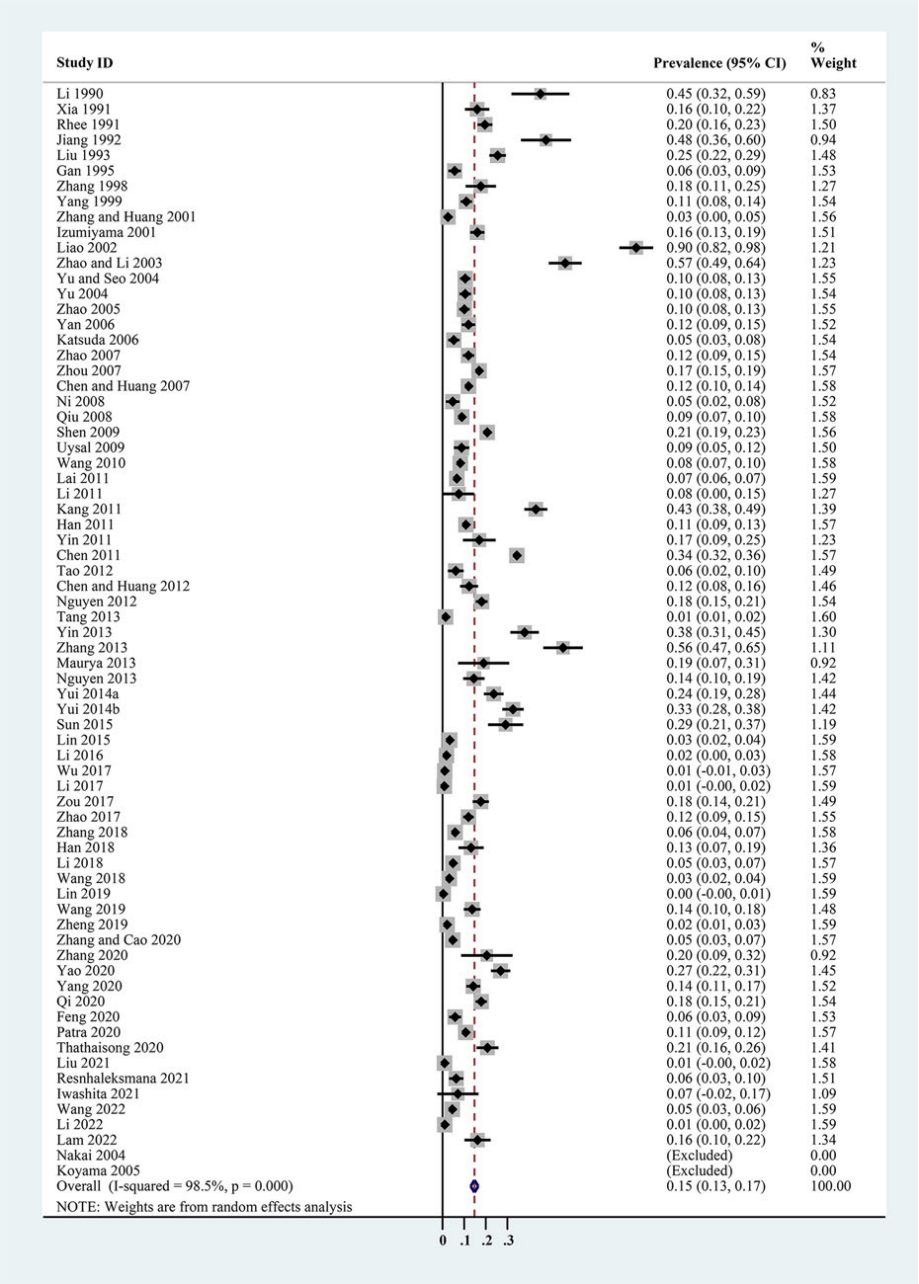
Forest plot of the prevalence estimates of *Cryptosporidium* infection in pigs in Asia.

**Fig. 4.**
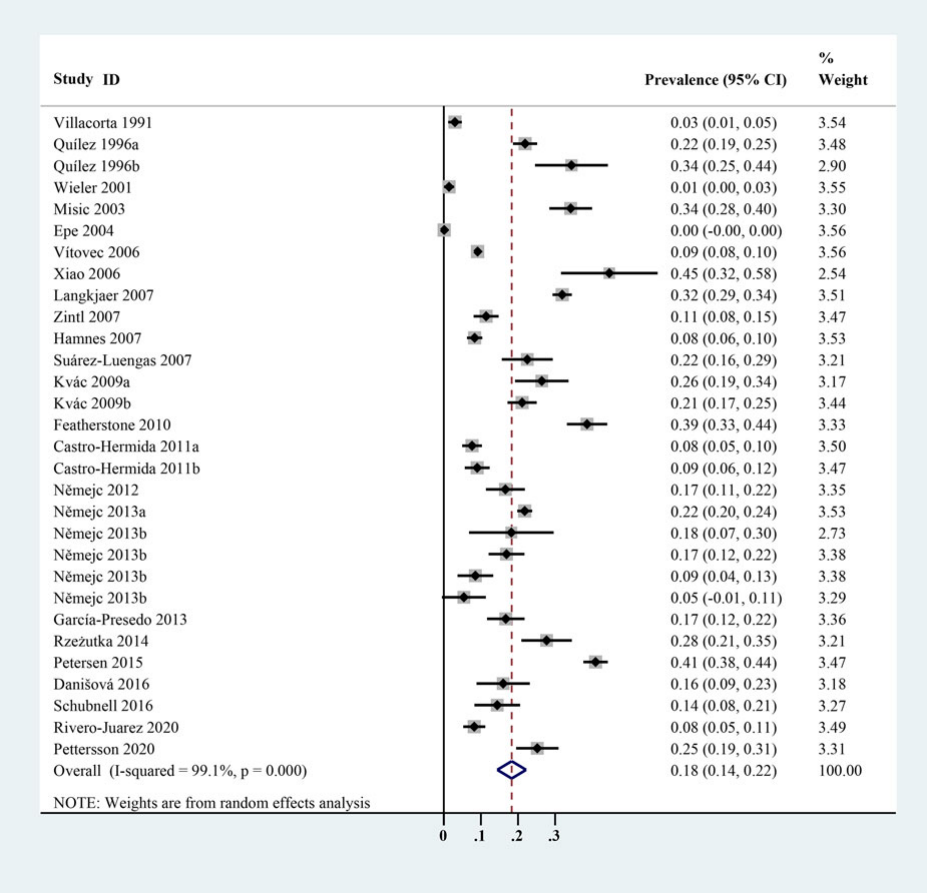
Forest plot of the prevalence estimates of *Cryptosporidium* infection in pigs in Europe.

**Fig. 5.**
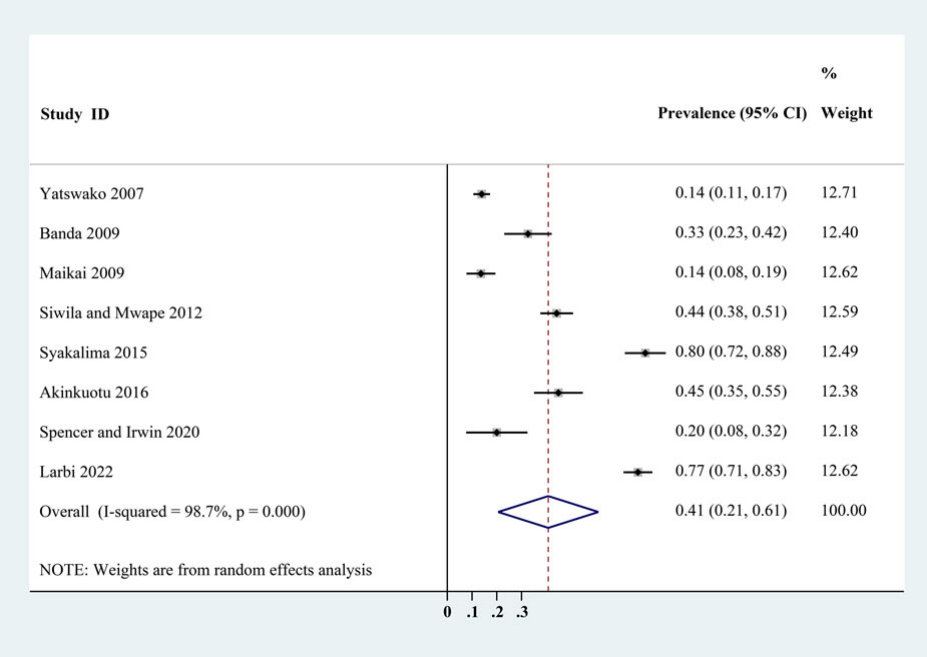
Forest plot of the prevalence estimates of *Cryptosporidium* infection in pigs in Africa.

**Fig. 6.**
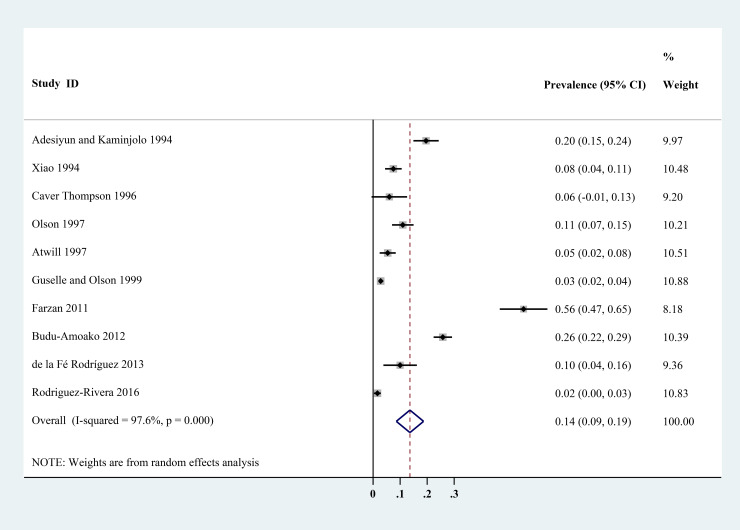
Forest plot of the prevalence estimates of *Cryptosporidium* infection in pigs in North America.

**Fig. 7.**
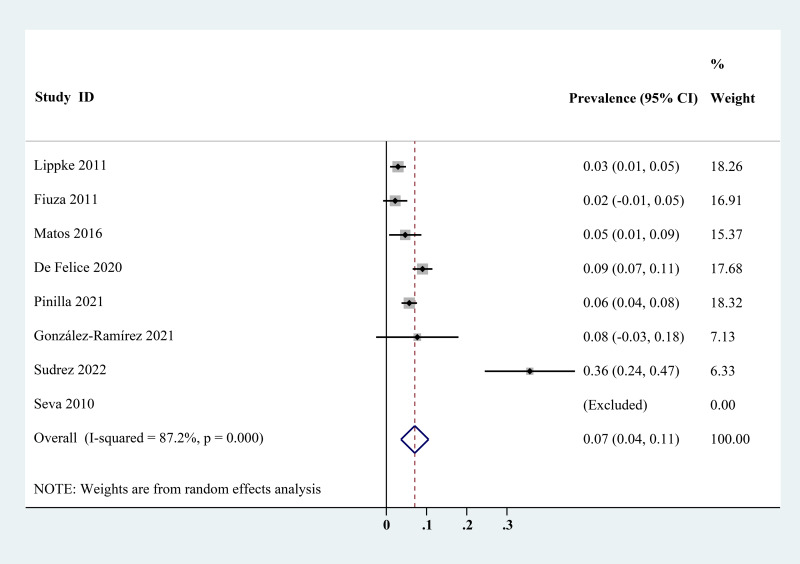
Forest plot of the prevalence estimates of *Cryptosporidium* infection in pigs in South America.

**Fig. 8.**
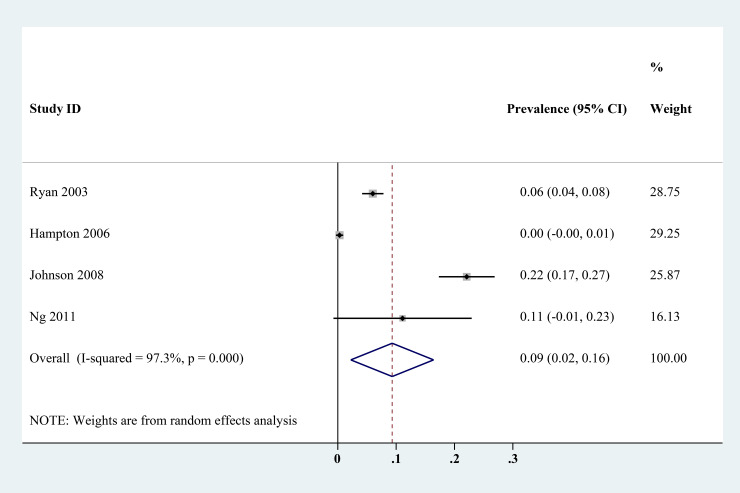
Forest plot of the prevalence estimates of *Cryptosporidium* infection in pigs in Oceania.

The estimated *Cryptosporidium* prevalence in pigs ranged from 7.1% [95% confidence interval (CI) 3.6–10.5%] to 40.8% (95% CI 20.6–61.0%), with substantial heterogeneity (*I*^2^ = 98.8%, *P* < 0.001). On a global scale, pooled estimated prevalence of *Cryptosporidium* infection in pigs was 16.3% (95% CI 15.0–17.6%, 8560/64 809) (Table 2). On 6 continents (Table 2, Figs 3–8), the infection rates of *Cryptosporidium* in pigs were 14.8% in Asia, 18.3% in Europe, 40.8% in Africa, 13.6% in North America, 7.1% in South America and 9.3% in Oceania. The highest number of studies on *Cryptosporidium* infections in pigs originated from Asia (*n* = 71). The highest prevalence rate was reported in South Africa [80.0% (95% CI 71.6–88.4%)], and the lowest prevalence rate was in Germany [0.4% (95% CI 0.1–0.6%)] (Table 1).

### Prevalence related to age, presence or absence of diarrhoea and *Cryptosporidium* species

The *Cryptosporidium* infection rate in post-weaned pigs was 25.8% (95% CI 21.8–29.8%, 2739/11 824). This was significantly higher than that in pre-weaned pigs [12.0%, 95% CI 9.9–14.0%, 1061/11 370, odds ratio (OR) 2.93, *P* < 0.05], fattening pigs (17.4%, 95% CI 14.8–20.0%, 1186/8815, OR 1.94, *P* < 0.05) and adult pigs (12.7%, 95% CI 10.4–15.1%, 980/9658, OR 2.67, *P* < 0.05) (Table 2). The infection rate for pigs with diarrhoea was 8.0% (95% CI 5.6–10.3%, 348/4874), while the infection rate for pigs without diarrhoea was 12.2% (95% CI 8.4–15.9%, 371/3501) (Table 2). Seven *Cryptosporidium* species (*C. scrofarum*, *C. suis*, *C. parvum*, *C. muris*, *C. tyzzeri*, *C. andersoni*, *C. struthioni*) were detected in pigs globally (Table 3). The prevalence rate of *C. scrofarum* was 7.9% (95% CI 6.9–8.8%, 1491/23 168) and that of *C. suis* was 4.7% (95% CI 3.8–5.6%, 1385/25 036) (Table 2). In Europe, *C. scrofarum* and *C. suis* infection rates were the highest, at 10.3% (678/6613) and 8.0% (881/10 951), respectively (Table S2).

### Prevalence according to geographic and climatic variables

We analysed geographic subgroup factors. The prevalence of *Cryptosporidium* in pigs in regions with a −30° to 0° latitude range (22.9%, 95% CI 8.3–37.5%, 193/872), 0°–60° longitude range (29.3%, 95% CI 17.9–40.7%, 774/5729), 5–10 °C mean yearly temperature (25.4%, 95% CI 16.3–34.6%, 603/4991), <60% mean yearly relative humidity (21.5%, 95% CI 15.0–28.0%, 627/3921), 800–1200 mm mean yearly precipitation (20.7%, 95% CI 15.5–25.9%, 2006/10 586) was higher than that in other regions (Table S3).

### Sensitivity analysis and publication bias

**Fig. 9.**
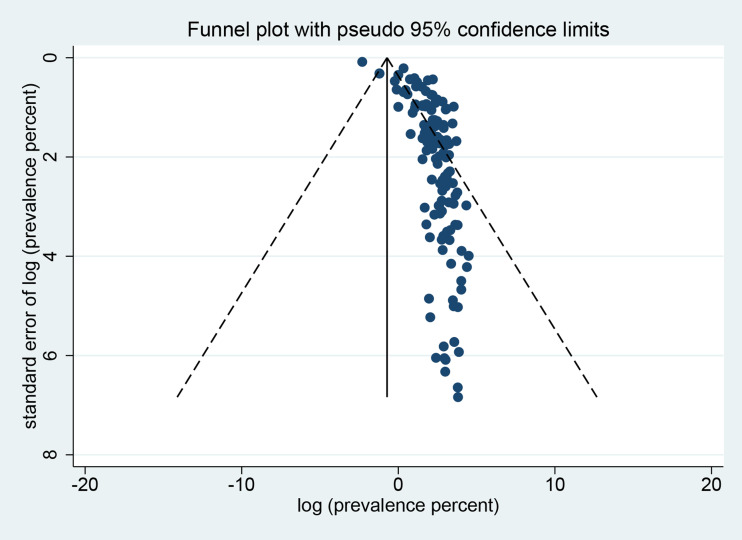
Funnel plot for examination of publication bias of the prevalence estimates of *Cryptosporidium* infection in pigs across the world.

Sensitivity analysis indicated that the analysis was reliable (Figs S1–S6). We often used a funnel plot to measure the publication bias in selected articles. Some points fell outside the funnel and the funnel plot showed obvious asymmetry (Fig. 9). The *P* value was less than 0.001 by Egger's test (Table S4), indicating that obvious publication bias was found.

### Sources of heterogeneity by meta-regression analysis

Univariate meta-regression analysis was used to determine the sources of heterogeneity. Age (*P* < 0.001), *Cryptosporidium* species (*P* = 0.002) and latitude (*P* = 0.028) were the factors that fostered heterogeneity. Region (*P* = 0.381), presence or absence of diarrhoea (*P* = 0.367), longitude (*P* = 0.793), mean temperature (*P* = 0.345), mean relative humidity (*P* = 0.356) and mean yearly precipitation (*P* = 0.548) were the factors that affected heterogeneity (Tables 2 and S3).

## Discussion

A meta-analysis based on selected datasets from 36 countries on 6 continents produced an estimate of *Cryptosporidium* prevalence in pigs. As mentioned in a previous systematic review, *Cryptosporidium* prevalence in pigs was the highest in Asia, Africa and Europe (Hatam-Nahavandi *et al*., 2019). Compared with previous study, the prevalence of *Cryptosporidium* in pigs was the highest in Africa, Europe and Asia in our study. In Europe, the highest infection rate was in the UK (38.6%, 95% CI 33.2–44.1%) (Featherstone *et al*., 2010), while the lowest rate was in Germany (0.4%, 95% CI 0.1–0.6%) (Wieler *et al*., 2001; Epe *et al*., 2004). *Cryptosporidium* infection in pigs differs between countries and also in different regions of the same country. In China, 1 study reported an infection rate of only 0.9% (2/216) in pigs in Zhejiang (Liu *et al*., 2021), while another study found a much higher infection rate of 26.9% (101/375) in pigs in Shaanxi (Yao *et al*., 2020).

Previous studies demonstrated that the rate of *Cryptosporidium* infection in pigs was related to age factors (Maddox-Hyttel *et al*., 2006; Featherstone *et al*., 2010). In our analysis, the *Cryptosporidium* infection rate in post-weaned pigs was significantly higher than that in pigs of other age groups. This is consistent with other studies (Wang *et al*., 2010; Yui *et al*., 2014*a*, 2014*b*; Petersen *et al*., 2015; Pettersson *et al*., 2020; Qi *et al*., 2020). Post-weaned piglets may be more susceptible to *Cryptosporidium* infection due to reduced immunity resulting from the loss of maternal immunity, or it may be due to weaning stress (Maddox-Hyttel *et al*., 2006; Li *et al*., 2018*b*). However, other studies revealed slightly divergent results. In Vietnam, the *Cryptosporidium* infection rate in pre-weaned pigs was higher (24.7%; 67/271) than that in post-weaned pigs (17.2%; 51/296), fattening pigs (7.1%; 7/98) or adult pigs (12.0%; 9/75) (Nguyen *et al*., 2012). In China, 2 studies showed higher rates of *Cryptosporidium* infection in finishing pigs than in pre-weaned, post-weaned and adult pigs (Chen and Huang, 2007; Wang *et al*., 2022). In general, *Cryptosporidium* infection in post-weaned pigs has attracted greater attention. However, high rates of *Cryptosporidium* infection in pigs of other age groups suggest that different management measures among the geographical areas may be involved in infection.

The global prevalence of *Cryptosporidium* infection in pigs without diarrhoea was higher than that in pigs suffering from diarrhoea (*P* < 0.05). Most of the articles did not mention the presence or absence of diarrhoea in pigs. Insufficient data collection may also affect the stability of the results. Therefore, the relationship between *Cryptosporidium* infection and diarrhoea in pigs remains unclear. Experimental infection studies showed that pigs shed a high number of *Cryptosporidium* oocysts but had no or mild diarrhoea. When *Cryptosporidium* was co-infected with other enteric pathogens, pigs exhibited significant diarrhoea and had a high mortality rate (Enemark *et al*., 2003). These results indicated that feces of apparently healthy pigs may also contain *Cryptosporidium* oocysts and that prevention of *Cryptosporidium* transmission in healthy pigs should be considered.

Pre-weaned pigs shed significantly more *Cryptosporidium* oocysts than older pigs, and this was associated with *C. suis* infection (Kvác *et al*., 2009*b*). Piglets were more susceptible to *C. suis* infection, while older pigs were more susceptible to *C. scrofarum* (Yin *et al*., 2013). Compared with previous studies, *C. suis* and *C. scrofarum* are still the dominant species in pigs. Other *Cryptosporidium* species (*C. parvum*, *C. muris*, *C. tyzzeri*, *C. andersoni*, *C. struthioni*) have occasionally been reported in pigs. House mice were the main hosts of *C. muris* and *C. tyzzeri* (Feng *et al*., 2018), and mice on pig farms may be involved in transmitting *Cryptosporidium*. *Cryptosporidium parvum* infection in pigs mainly occurred in Europe (Wieler *et al*., 2001; Zintl *et al*., 2007; Kvác *et al*., 2009*a*; García-Presedo *et al*., 2013; Němejc *et al*., 2013*a*; Rzeżutka *et al*., 2014; Pettersson *et al*., 2020), Asia (Katsuda *et al*., 2006; Qi *et al*., 2020; Yao *et al*., 2020; Liu *et al*., 2021; Resnhaleksmana *et al*., 2021) and North America (Atwill *et al*., 1997; Farzan *et al*., 2011; Budu-Amoako *et al*., 2012). *Cryptosporidium parvum* may play a role in zoonotic transmission on pig farms. Therefore, necessary measures should be taken to reduce contact between breeders and pigs to reduce the transmission of *Cryptosporidium* from pigs to humans.

Oocysts can survive for a long time under many environmental conditions (Rose *et al*., 2002; Gorospe, 2005; Alum *et al*., 2014), and a single oocyst is sufficient to infect and cause disease in a susceptible host (Ramirez *et al*., 2004). The prevalence of *Cryptosporidium* in pigs in regions with −30° to 0° latitude range (22.9%, 193/872) and 0°–60° longitude range (29.3%, 774/5729) was higher than that in pigs in other regions. Jagai *et al*. predicted that climate change would increase the spread of cryptosporidiosis infection, and that this spread would vary by season and location (Jagai *et al*., 2009). The prevalence of *Cryptosporidium* in pigs was higher in areas with a mean yearly precipitation of 800–1200 mm (20.7%, 2006/10 586), mean yearly temperature of 5–10 °C (25.4%, 603/4991) and mean yearly relative humidity of < 60% (21.5%, 627/3921). These results indicated that cryptosporidiosis was more likely to occur in warm and rainy areas. Factors such as rainfall, temperature and humidity influence the life cycle of *Cryptosporidium* and may influence the timing and intensity of disease outbreaks (Patz *et al*., 2000).

### Limitations

The current study has the following limitations:

1. Some countries had only 1 publication of *Cryptosporidium* infecting pigs in the past 30 years.
2. Unpublished data were not included in the analysis.
3. Data of some conference abstracts were not included in the analysis.
4. Some publications lacked full text, and these articles were excluded.
5. Analysis of the factors involved was limited. Factors such as season, feeding model and pig breed may also be sources of heterogeneity.

Even so, we believe that the results of this study are close to the true global prevalence of *Cryptosporidium* in pigs.

## Conclusions

This analysis shows that *Cryptosporidium* infection in pigs is widespread worldwide. *Cryptosporidium* can cause high levels of disease, particularly in Africa where infection rates are as high as 40.8%. *Cryptosporidium suis* is the dominant species in pre-weaned pigs while *C. scrofarum* is the dominant species in fattening and adult pigs. Pig age is an important risk factor associated with cryptosporidiosis. Age should be considered so that farmers can implement effective management plans based on geographical area and environmental factors and prevent zoonotic transmission. These findings highlight the role of pigs as possible potential hosts of zoonotic cryptosporidiosis and the need for additional studies on the prevalence, transmission and control of *Cryptosporidium* in pigs.

## Data Availability

All data generated or used during the study appear in the submitted article.

## Supplementary material

For supplementary material accompanying this paper visit https://doi.org/10.1017/S0031182023000276. click here to view supplementary material
