# Supplementary material for: Global prevalence of Cryptosporidium spp. in pigs: a systematic review and meta-analysis
Source: Parasitology. 2023 Mar 20;150(6):531–44. doi: 10.1017/S0031182023000276 (PMC10260304; doi:10.1017/S0031182023000276)
Supplement: Supplementary file 1 [file S0031182023000276sup.zip › S0031182023000276sup001.docx]

**Supplemental materials**

**Journal:** Parasitology

**Title:** Global prevalence of *Cryptosporidium* spp. in pigs: a systematic review and meta-analysis

Yuancai Chen^1 †^, Huikai Qin^1 †^, Yayun Wu^1^, Jianying Huang^1^, Junqiang Li^1^, Longxian Zhang^1,*^

^1^ College of Veterinary Medicine, Henan Agricultural University, Zhengzhou 450002, P. R. China

* **Corresponding author:** Longxian Zhang, College of Veterinary Medicine, Henan Agricultural University, No. 15 Longzihu University Area, Zhengdong New District, Zhengzhou 450046, China.

Tel: 86-371-56990163; Fax: 86-371-56990163;

E-mail: [zhanglx8999@henau.edu.cn](mailto:zhanglx8999@henau.edu.cn)

**Table S4** Egger’ s test for publication bias.

| Std_Eff | Coef. | Std. Err. | t | P>\|t\| | 95% Conf. Interval |
| --- | --- | --- | --- | --- | --- |
| slope | -0.556 | 0.426 | -1.30 | 0.194 | -1.400 to 0.288 |
| bias | 8.464 | 0.632 | 13.39 | 0.000 | 7.213–9.714 |
