# Supplementary material for: Global prevalence of Cryptosporidium spp. in pigs: a systematic review and meta-analysis
Source: Parasitology. 2023 Mar 20;150(6):531–44. doi: 10.1017/S0031182023000276 (PMC10260304; doi:10.1017/S0031182023000276)
Supplement: Supplementary file 1 [file S0031182023000276sup.zip › S0031182023000276sup003.docx]

**Supplemental materials**

**Journal:** Parasitology

**Title:** Global prevalence of *Cryptosporidium* spp. in pigs: a systematic review and meta-analysis

Yuancai Chen^1 †^, Huikai Qin^1 †^, Yayun Wu^1^, Jianying Huang^1^, Junqiang Li^1^, Longxian Zhang^1,*^

^1^ College of Veterinary Medicine, Henan Agricultural University, Zhengzhou 450002, P. R. China

* **Corresponding author:** Longxian Zhang, College of Veterinary Medicine, Henan Agricultural University, No. 15 Longzihu University Area, Zhengdong New District, Zhengzhou 450046, China.

Tel: 86-371-56990163; Fax: 86-371-56990163;

E-mail: [zhanglx8999@henau.edu.cn](mailto:zhanglx8999@henau.edu.cn)

**Table S2** Studies included and quality scores in the analysis

| Study ID | Country | Sampling time | Detection method | Total samples | Positive samples | Prevalence | Purpose clearly or not | Three or more risk factors or not | Sampled method detailedly or not | Sampled time clearly or not | The number of samples is ≥ 200 or not | Score | Study Quality |
| --- | --- | --- | --- | --- | --- | --- | --- | --- | --- | --- | --- | --- | --- |
| **Asia (71)** |  |  |  |  |  |  |  |  |  |  |  |  |  |
| Li 1990 | China | UN | Microscopy | 53 | 24 | 0.453 | Y | N | Y | N | N | 2 | Middle |
| Xia 1991 | China | 1988.10-1989.06 | Microscopy | 162 | 26 | 0.160 | Y | N | Y | Y | N | 3 | Middle |
| Jiang 1992 | China | UN | Microscopy | 71 | 34 | 0.479 | Y | N | Y | N | N | 2 | Middle |
| Liu 1993 | China | 1989-1990 | Microscopy | 495 | 126 | 0.255 | Y | N | Y | Y | Y | 4 | High |
| Gan 1995 | China | 1992.05-10 | Microscopy | 233 | 13 | 0.056 | Y | N | Y | Y | Y | 4 | High |
| Zhang 1998 | China | UN | Microscopy | 113 | 20 | 0.177 | Y | N | Y | N | N | 2 | Middle |
| Yang 1999 | China | 1994-1996 | Microscopy | 527 | 58 | 0.110 | N | N | Y | Y | Y | 3 | Middle |
| Zhang and Huang 2001 | China | 1999.07-2000.04 | Microscopy | 198 | 5 | 0.025 | Y | Y | Y | Y | N | 4 | High |
| Liao 2002 | China | UN | Microscopy | 58 | 52 | 0.897 | N | N | Y | N | N | 1 | Low |
| Zhao and Li 2003 | China | 2001.11-2002.05 | Microscopy | 162 | 92 | 0.568 | Y | N | Y | Y | N | 3 | Middle |
| Zhao 2005 | China | UN | Microscopy | 577 | 58 | 0.101 | Y | Y | Y | N | Y | 4 | High |
| Yan 2006 | China | 2003.07-2004.02 | Microscopy | 417 | 50 | 0.120 | Y | N | Y | Y | Y | 4 | High |
| Zhao 2007 | China | UN | Microscopy | 577 | 69 | 0.120 | Y | Y | Y | N | Y | 4 | High |
| Zhou 2007 | China | 2005.10-2006.12 | Microscopy | 1790 | 303 | 0.169 | Y | Y | Y | Y | Y | 5 | High |
| Chen and Huang 2007 | China | 2005.08-2006.07 | PCR | 1488 | 178 | 0.120 | Y | Y | Y | Y | Y | 5 | High |
| Ni 2008 | China | UN | Microscopy | 170 | 8 | 0.047 | N | N | Y | N | N | 1 | Low |
| Qiu 2008 | China | UN | Microscopy | 1429 | 127 | 0.089 | Y | N | Y | N | Y | 3 | Middle |
| Shen 2009 | China | UN | Microscopy | 1510 | 312 | 0.207 | Y | Y | Y | N | Y | 4 | High |
| Wang 2010 | China | 2006.10-2007.08 | PCR | 1350 | 111 | 0.082 | Y | Y | Y | Y | Y | 5 | High |
| Lai 2011 | China | 2007.09-2009.09 | Microscopy | 2971 | 196 | 0.066 | Y | Y | Y | Y | Y | 5 | High |
| Li 2011 | China | UN | Microscopy | 53 | 4 | 0.075 | Y | N | Y | N | N | 2 | Middle |
| Kang 2011 | China | 2011 | Microscopy | 333 | 144 | 0.432 | N | N | Y | Y | Y | 3 | Middle |
| Han 2011 | China | UN | Microscopy | 1210 | 131 | 0.108 | Y | N | Y | N | Y | 3 | Middle |
| Yin 2011 | China | 2009.04-10 | PCR | 94 | 16 | 0.170 | Y | N | Y | Y | N | 3 | Middle |
| Chen 2011 | China | 2006.06-2009.09 | PCR | 2323 | 800 | 0.344 | Y | Y | Y | Y | Y | 5 | High |
| Tao 2012 | China | 2011.04-05 | Microscopy | 164 | 10 | 0.061 | Y | N | Y | Y | N | 3 | Middle |
| Chen and Huang 2012 | China | 2005.10-2007.06 | Microscopy | 236 | 29 | 0.123 | N | N | Y | Y | Y | 3 | Middle |
| Tang 2013 | China | UN | Microscopy | 3021 | 43 | 0.014 | Y | Y | Y | N | Y | 4 | High |
| Yin 2013 | China | 2009.04-2011.07 | PCR | 208 | 79 | 0.380 | Y | Y | Y | Y | Y | 5 | High |
| Zhang 2013 | China | 2011.10-2012.10 | PCR | 113 | 63 | 0.558 | Y | N | Y | Y | N | 3 | Middle |
| Sun 2015 | China | 2012.12-2014.12 | Microscopy | 120 | 35 | 0.292 | N | N | Y | Y | N | 2 | Middle |
| Lin 2015 | China | 2011.05-2014.05 | PCR | 1337 | 44 | 0.033 | Y | Y | Y | Y | Y | 5 | High |
| Li 2016 | China | 2014-2015 | PCR | 326 | 6 | 0.018 | Y | N | Y | Y | Y | 4 | High |
| Wu 2017 | China | 2016.06-07 | Microscopy | 101 | 1 | 0.010 | N | N | Y | Y | N | 2 | Middle |
| Li 2017 | China | 2014-2015 | PCR | 357 | 3 | 0.008 | Y | N | Y | Y | Y | 4 | High |
| Zou 2017 | China | 2016 | PCR | 396 | 70 | 0.177 | Y | Y | Y | Y | Y | 5 | High |
| Zhao 2017 | China | UN | PCR | 648 | 78 | 0.120 | Y | N | Y | N | Y | 3 | Middle |
| Zhang 2018 | China | 2016.05-2017.08 | Microscopy | 1206 | 70 | 0.058 | Y | Y | Y | Y | Y | 5 | High |
| Han 2018 | China | 2016.11-2017.01 | PCR | 129 | 17 | 0.132 | Y | N | Y | Y | N | 3 | Middle |
| Li 2018 | China | 2014.10-12 | PCR | 500 | 24 | 0.048 | Y | Y | Y | Y | Y | 4 | High |
| Wang 2018 | China | 2016.01-09 | PCR | 897 | 28 | 0.031 | Y | N | Y | Y | Y | 4 | High |
| Lin 2019 | China | 2017.07-2018.06 | Microscopy | 300 | 1 | 0.003 | Y | N | Y | Y | Y | 4 | High |
| Wang 2019 | China | UN | PCR | 300 | 41 | 0.137 | Y | N | Y | N | Y | 3 | Middle |
| Zheng 2019 | China | 2016.03-06 | PCR | 1089 | 23 | 0.021 | Y | Y | Y | Y | Y | 5 | High |
| Zhang and Cao 2020 | China | 2018.03-2019.05 | Microscopy | 473 | 22 | 0.047 | Y | N | Y | Y | Y | 4 | High |
| Zhang 2020 | China | 2018.08 | PCR | 44 | 9 | 0.205 | Y | N | Y | Y | N | 3 | Middle |
| Yao 2020 | China | UN | PCR | 375 | 101 | 0.269 | Y | Y | Y | N | Y | 4 | High |
| Yang 2020 | China | UN | PCR | 450 | 64 | 0.142 | Y | N | Y | N | Y | 3 | Middle |
| Qi 2020 | China | 2017.09-2018.06 | PCR | 801 | 143 | 0.179 | Y | Y | Y | Y | Y | 5 | High |
| Feng 2020 | China | 2015.09-2018.11 | PCR | 257 | 15 | 0.058 | Y | N | Y | Y | Y | 4 | High |
| Liu 2021 | China | 2019.11 | PCR | 216 | 2 | 0.009 | Y | N | Y | Y | Y | 4 | High |
| Wang 2022 | China | UN | PCR | 1254 | 57 | 0.045 | Y | Y | Y | N | Y | 4 | High |
| Li 2022 | China | 2019.09-12 | PCR | 826 | 8 | 0.010 | Y | Y | Y | Y | Y | 5 | High |
| Lam 2022 | China | UN | PCR | 142 | 23 | 0.162 | N | Y | Y | N | N | 2 | Middle |
| Maurya 2013 | India | 2009-2012 | PCR | 42 | 8 | 0.190 | N | Y | Y | Y | N | 3 | Middle |
| Patra 2020 | India | 2017.01-2018.12 | Microscopy | 1153 | 123 | 0.107 | Y | N | Y | Y | Y | 4 | High |
| Resnhaleksmana 2021 | Indonesia | UN | PCR | 205 | 13 | 0.063 | Y | N | Y | N | Y | 3 | Middle |
| Izumiyama 2001 | Japan | 1998.06-2000.06 | Microscopy | 484 | 78 | 0.161 | Y | N | Y | Y | Y | 4 | High |
| Nakai 2004 | Japan | 1995-1997 | PCR | 500 | 0 | 0.000 | N | N | Y | Y | Y | 3 | Middle |
| Koyama 2005 | Japan | 2001.09 | PCR | 108 | 0 | 0.000 | N | N | Y | Y | N | 2 | Middle |
| Katsuda 2006 | Japan | 2001.10-2003.09 | PCR | 269 | 14 | 0.052 | Y | N | Y | Y | Y | 4 | High |
| Yui 2014b | Japan | 2010.01-2011.01 | PCR | 344 | 112 | 0.326 | Y | N | Y | Y | Y | 4 | High |
| Yui 2014a | Japan | 2009.09-11 | PCR | 334 | 79 | 0.237 | Y | N | Y | Y | Y | 4 | High |
| Rhee 1991 | Korea | UN | Microscopy | 500 | 98 | 0.196 | N | N | Y | N | Y | 2 | Middle |
| Yu and Seo 2004 | Korea | 2000.11-2001.06 | Microscopy | 589 | 62 | 0.105 | Y | N | Y | Y | Y | 4 | High |
| Yu 2004 | Korea | 2001.09-2002.06 | Microscopy | 493 | 52 | 0.105 | N | N | Y | Y | Y | 3 | Middle |
| Thathaisong 2020 | Thailand | 2015.05-2016.01 | PCR | 245 | 51 | 0.208 | Y | Y | Y | Y | Y | 5 | High |
| Uysal 2009 | Turkey | UN | Microscopy | 238 | 21 | 0.088 | Y | N | Y | N | Y | 3 | Middle |
| Nguyen 2012 | Vietnam | 2009.02-12 | Microscopy | 740 | 134 | 0.181 | Y | Y | Y | Y | Y | 5 | High |
| Nguyen 2013 | Vietnam | 2009.10-2010.01 | PCR | 193 | 28 | 0.145 | Y | N | Y | Y | N | 3 | Middle |
| Iwashita 2021 | Vietnam | 2015.08-10 | PCR | 28 | 2 | 0.071 | N | N | Y | Y | N | 2 | Middle |
| **Europe (30)** |  |  |  |  |  |  |  |  |  |  |  |  |  |
| Němejc 2013b | Austria | 2011-2012 | PCR | 44 | 8 | 0.182 | Y | N | Y | Y | N | 3 | Middle |
| Vítovec 2006 | Czech Republic | 2002-2004 | PCR | 4338 | 394 | 0.091 | Y | N | Y | Y | Y | 4 | High |
| Kvác 2009a | Czech Republic | UN | PCR | 144 | 38 | 0.264 | Y | N | Y | N | N | 2 | Middle |
| Kvác 2009b | Czech Republic | 2007 | PCR | 413 | 87 | 0.211 | Y | Y | Y | Y | Y | 5 | High |
| Němejc 2012 | Czech Republic | 2009-2010 | PCR | 193 | 32 | 0.165 | Y | N | Y | Y | N | 3 | Middle |
| Němejc 2013b | Czech Republic | 2011-2012 | PCR | 231 | 39 | 0.169 | Y | N | Y | Y | Y | 4 | High |
| Němejc 2013a | Czech Republic | 2009-2011 | PCR | 1620 | 353 | 0.218 | Y | N | Y | Y | Y | 4 | High |
| Langkjaer 2007 | Denmark | UN | PCR | 1237 | 395 | 0.319 | Y | N | Y | N | Y | 3 | Middle |
| Petersen 2015 | Denmark | 2011.09-2012.06 | PCR | 856 | 350 | 0.409 | Y | Y | Y | Y | Y | 5 | High |
| Wieler 2001 | Germany | UN | PCR | 287 | 4 | 0.014 | Y | N | Y | N | Y | 3 | Middle |
| Epe 2004 | Germany | 1998-2002 | Microscopy | 1427 | 2 | 0.001 | N | N | Y | Y | Y | 3 | Middle |
| Zintl 2007 | Ireland | 2005.05-07 | PCR | 342 | 39 | 0.114 | Y | N | Y | Y | Y | 4 | High |
| Hamnes 2007 | Norway | 2004.03-2005.10 | PCR | 684 | 57 | 0.083 | Y | N | Y | Y | Y | 4 | High |
| Němejc 2013b | Poland | 2011-2012 | PCR | 129 | 11 | 0.085 | Y | N | Y | Y | N | 3 | Middle |
| Rzeżutka 2014 | Poland | 2008.05-09 | PCR | 166 | 46 | 0.277 | Y | Y | Y | Y | N | 4 | High |
| Misic 2003 | Serbia | UN | Microscopy | 260 | 89 | 0.342 | Y | N | Y | N | Y | 3 | Middle |
| Němejc 2013b | Slovak Republic | 2011-2012 | PCR | 56 | 3 | 0.054 | Y | N | Y | Y | N | 3 | Middle |
| Danišová 2016 | Slovak Republic | 2013.09-2014.09 | PCR | 100 | 16 | 0.160 | N | N | Y | Y | N | 2 | Middle |
| Villacorta 1991 | Spain | 1987.01-1989.06 | Microscopy | 329 | 10 | 0.030 | N | N | Y | Y | Y | 3 | Middle |
| Quílez 1996a | Spain | 1992.01-1994.01 | Microscopy | 620 | 136 | 0.219 | Y | N | Y | N | Y | 3 | Middle |
| Quílez 1996b | Spain | 1990-1994 | Microscopy and IFA | 90 | 31 | 0.344 | N | N | Y | Y | N | 2 | Middle |
| Suárez-Luengas 2007 | Spain | 2004.03-06 | PCR | 142 | 32 | 0.225 | Y | N | Y | Y | N | 3 | Middle |
| Castro-Hermida 2011a | Spain | 2008.08-2009.01 | IFA | 381 | 29 | 0.076 | Y | N | Y | Y | Y | 4 | High |
| Castro-Hermida 2011b | Spain | 2008-2009 | PCR | 279 | 25 | 0.090 | N | N | Y | Y | Y | 3 | Middle |
| García-Presedo 2013 | Spain | 2009-2010 | PCR | 209 | 35 | 0.167 | Y | N | Y | Y | Y | 4 | High |
| Rivero-Juarez 2020 | Spain | 2015-2016 | PCR | 328 | 27 | 0.082 | Y | N | Y | Y | Y | 4 | High |
| Pettersson 2020 | Sweden | 2017.10-2018.10 | PCR | 222 | 56 | 0.252 | Y | N | Y | Y | Y | 4 | High |
| Schubnell 2016 | Switzerland | 2014.01-12 | PCR | 125 | 18 | 0.144 | Y | N | Y | Y | N | 3 | Middle |
| Featherstone 2010 | UK | 2007.01-12 | IFA | 308 | 119 | 0.386 | Y | N | Y | Y | Y | 4 | High |
| Xiao 2006 | UK\Ireland | 2002-2004 | PCR | 56 | 25 | 0.446 | Y | N | Y | Y | N | 3 | Middle |
| **Africa (8)** |  |  |  |  |  |  |  |  |  |  |  |  |  |
| Larbi 2022 | Ghana | UN | Microscopy | 200 | 154 | 0.770 | Y | N | Y | N | Y | 3 | Middle |
| Banda 2009 | Malawi | 2001.10-2003.05 | PCR | 92 | 30 | 0.326 | N | N | Y | Y | N | 2 | Middle |
| Spencer and Irwin 2020 | Madagascar | 2014.05-07 | Microscopy | 40 | 8 | 0.200 | N | N | Y | Y | N | 2 | Middle |
| Yatswako 2007 | Nigeria | 2005.11-2006.07 | Microscopy | 402 | 56 | 0.139 | Y | N | Y | Y | Y | 4 | High |
| Maikai 2009 | Nigeria | 2008.07-09 | Microscopy | 132 | 18 | 0.136 | Y | Y | Y | Y | N | 4 | High |
| Akinkuotu 2016 | Nigeria | 2012 | ELISA | 98 | 44 | 0.449 | Y | N | Y | Y | N | 3 | Middle |
| Syakalima 2015 | South Africa | 2013.07-11 | ELISA | 90 | 72 | 0.800 | Y | N | Y | Y | N | 3 | Middle |
| Siwila and Mwape 2012 | Zambia | 2011.03-06 | Microscopy and IFA | 217 | 96 | 0.442 | Y | N | Y | Y | Y | 4 | High |
| **North America (10)** |  |  |  |  |  |  |  |  |  |  |  |  |  |
| Olson 1997 | Canada | 1995.06-08 | Microscopy | 236 | 26 | 0.110 | N | N | Y | Y | Y | 3 | Middle |
| Guselle and Olson 1999 | Canada | UN | UN | 1602 | 45 | 0.028 | Y | N | N | N | Y | 2 | Middle |
| Farzan 2011 | Canada | 2005.09-2006.05 | PCR | 122 | 68 | 0.557 | Y | N | Y | Y | N | 3 | Middle |
| Budu-Amoako 2012 | Canada | 2007.02-11 | PCR | 633 | 163 | 0.258 | Y | N | Y | Y | Y | 4 | High |
| Adesiyun and Kaminjolo 1994 | Trinidad | 1992.03-08 | Microscopy | 275 | 54 | 0.196 | N | N | Y | Y | Y | 3 | Middle |
| Xiao 1994 | USA | 1993.02-03 | IFA | 281 | 21 | 0.075 | Y | N | Y | Y | Y | 4 | High |
| Caver 1996 | USA | 1992.08-1994.05 | Microscopy | 50 | 3 | 0.060 | N | N | Y | Y | N | 2 | Middle |
| Atwill 1997 | USA | 1995.06-09 | PCR | 221 | 12 | 0.054 | Y | Y | Y | Y | Y | 5 | High |
| Rodriguez-Rivera 2016 | USA | 2014.02-2015.05 | PCR | 370 | 6 | 0.016 | Y | N | Y | Y | Y | 4 | High |
| de la Fé Rodríguez 2013 | Cuba | 2008.05-06 | ELISA | 90 | 9 | 0.100 | Y | N | Y | Y | N | 3 | Middle |
| **South America (8)** |  |  |  |  |  |  |  |  |  |  |  |  |  |
| Argentina | De Felice 2020 | UN | PCR | 520 | 47 | 0.090 | Y | N | Y | N | Y | 3 | Middle |
| Brazil | Seva 2010 | 2007.06 | PCR | 25 | 0 | 0.000 | N | N | Y | Y | N | 2 | Middle |
| Brazil | Lippke 2011 | 2007.05-09 | Microscopy | 276 | 8 | 0.029 | Y | N | Y | Y | Y | 4 | High |
| Brazil | Fiuza 2011 | UN | PCR | 91 | 2 | 0.022 | Y | N | Y | N | N | 2 | Middle |
| Brazil | Matos 2016 | 2007-2008 | Microscopy | 107 | 5 | 0.047 | Y | N | Y | Y | N | 3 | Middle |
| Colombia | Pinilla 2021 | 2019.09-12 | ELISA | 558 | 32 | 0.057 | Y | Y | Y | Y | Y | 5 | High |
| Colombia | Sudrez 2022 | 2018.04-10 | Microscopy | 70 | 25 | 0.357 | Y | N | Y | Y | N | 3 | Middle |
| Ecuador | González-  Ramírez 2021 | 2019.06-07 | Microscopy | 26 | 2 | 0.077 | N | N | Y | Y | N | 2 | Middle |
| **Oceania (4)** |  |  |  |  |  |  |  |  |  |  |  |  |  |
| Australia | Ryan 2003 | 1999.08-2001.07 | PCR | 646 | 39 | 0.060 | Y | N | Y | Y | Y | 4 | High |
| Australia | Hampton 2006 | 2001.05-2003.07 | PCR | 292 | 1 | 0.003 | Y | N | Y | Y | Y | 4 | High |
| Australia | Johnson 2008 | 2005.08-11 and 2006.03-08 | PCR | 289 | 64 | 0.221 | Y | N | Y | Y | Y | 4 | High |
| Australia | Ng 2011 | UN | PCR | 27 | 3 | 0.111 | N | N | Y | N | N | 1 | Low |

IFA: indirect immunoinfluscent assay; PCR: polymerase chain reaction; ELISA: enzyme-linked immunosorbent assay; UN: unknown
