# Supplementary material for: Global prevalence of Cryptosporidium spp. in pigs: a systematic review and meta-analysis
Source: Parasitology. 2023 Mar 20;150(6):531–44. doi: 10.1017/S0031182023000276 (PMC10260304; doi:10.1017/S0031182023000276)
Supplement: Supplementary file 1 [file S0031182023000276sup.zip › S0031182023000276sup004.docx]

**Supplemental materials**

**Journal:** Parasitology

**Title:** Global prevalence of *Cryptosporidium* spp. in pigs: a systematic review and meta-analysis

Yuancai Chen^1 †^, Huikai Qin^1 †^, Yayun Wu^1^, Jianying Huang^1^, Junqiang Li^1^, Longxian Zhang^1,*^

^1^ College of Veterinary Medicine, Henan Agricultural University, Zhengzhou 450002, P. R. China

* **Corresponding author:** Longxian Zhang, College of Veterinary Medicine, Henan Agricultural University, No. 15 Longzihu University Area, Zhengdong New District, Zhengzhou 450046, China.

Tel: 86-371-56990163; Fax: 86-371-56990163;

E-mail: [zhanglx8999@henau.edu.cn](mailto:zhanglx8999@henau.edu.cn)

**Table S3** Prevalence of *Cryptosporidium* based on geographical factors of pigs worldwide.

|  | **Number of**  **datasets** | **Total samples** | **Positive samples** | **Prevalence**  **% (95% CI)** | **Heterogeneity** | | | **Univariate meta-regression** | | **Correlation Analysis** |
| --- | --- | --- | --- | --- | --- | --- | --- | --- | --- | --- |
|  |  |  |  |  | **χ^2^** | ***P* value** | ***I*^2^** | ***P* value** | **Coefficient (95% CI)** | **Adj R^2^** |
| **Latitude (°)** |  |  |  |  |  |  |  | 0.028 | 0.744 (0.081–1.407) | 5.06% |
| -30 to 0 | 8 | 872 | 193 | 22.9 (8.3–37.5) | 452.55 | < 0.001 | 98.7% |  |  |  |
| 0*–*30 | 20 | 12219 | 1283 | 15.2 (11.8–18.5) | 1621.34 | < 0.001 | 98.8% |  |  |  |
| 30*–*60 | 49 | 28338 | 3781 | 16.3 (13.9–18.6) | 3335.71 | < 0.001 | 98.6% |  |  |  |
| **Longitude (°)** |  |  |  |  |  |  |  | 0.793 | -0.117 (-1.006 to 0.771) | -1.26% |
| < -60 | 8 | 2301 | 313 | 14.2 (7.4–21.1) | 299.98 | < 0.001 | 97.7% |  |  |  |
| -60 to 0 | 10 | 2205 | 387 | 17.7 (7.9–27.5) | 741.62 | < 0.001 | 98.9% |  |  |  |
| 0*–*60 | 8 | 5729 | 774 | 29.3 (17.9–40.7) | 469.53 | < 0.001 | 98.5% |  |  |  |
| 60*–*120 | 41 | 24611 | 2361 | 13.6 (11.6–15.5) | 2382.15 | < 0.001 | 98.3% |  |  |  |
| > 120 | 10 | 6583 | 1422 | 18.0 (9.6–26.4) | 1063.57 | < 0.001 | 99.2% |  |  |  |
| **Mean yearly temperature (****℃)** |  |  |  |  |  |  |  | 0.345 | 0.447 (-0.491 to 1.384) | -0.13% |
| 5–10 | 7 | 4991 | 603 | 25.4 (16.3–34.6) | 319.71 | < 0.001 | 98.1% |  |  |  |
| 10–15 | 13 | 4914 | 837 | 18.2 (13.0–23.4) | 373.09 | < 0.001 | 96.8% |  |  |  |
| 15–20 | 40 | 27824 | 3205 | 14.4 (12.1–16.6) | 3559.38 | < 0.001 | 98.9% |  |  |  |
| 20–25 | 9 | 2072 | 321 | 16.1 (9.2–23.0) | 287.09 | < 0.001 | 97.2% |  |  |  |
| > 25 | 8 | 1628 | 291 | 18.7 (5.8–31.5) | 675.52 | < 0.001 | 99.1% |  |  |  |
| **Mean yearly relative humidity (%)** |  |  |  |  |  |  |  | 0.356 | -0.234 (-0.363 to 1.076) | -0.04% |
| < 60 | 13 | 3921 | 627 | 21.5 (15.0–28.0) | 616.41 | < 0.001 | 98.1% |  |  |  |
| 60–70 | 22 | 13846 | 2079 | 14.8 (11.0–18.7) | 1435.84 | < 0.001 | 98.5% |  |  |  |
| 70–80 | 30 | 12843 | 1255 | 15.2 (12.6–17.8) | 2140.20 | < 0.001 | 98.7% |  |  |  |
| 80–90 | 12 | 10819 | 1296 | 17.7 (13.2–22.3) | 693.15 | < 0.001 | 98.4% |  |  |  |
| **Mean yearly precipitation (mm)** |  |  |  |  |  |  |  | 0.548 | -0.234 (-1.007 to 0.539) | -0.86% |
| 0–400 | 11 | 3485 | 379 | 10.4 (6.7–14.1) | 183.69 | < 0.001 | 94.6% |  |  |  |
| 400–800 | 21 | 14375 | 1576 | 18.4 (15.1–21.6) | 1050.58 | < 0.001 | 98.1% |  |  |  |
| 800–1200 | 22 | 10586 | 2006 | 20.7 (15.5–25.9) | 2883.63 | < 0.001 | 99.3% |  |  |  |
| 1200–1600 | 13 | 11007 | 1060 | 12.7 (9.4–16.0) | 1028.94 | < 0.001 | 98.8% |  |  |  |
| > 1600 | 10 | 1976 | 236 | 13.8 (8.7–19.0) | 119.17 | < 0.001 | 93.3% |  |  |  |
